# Supplementary material for: Site-specific risk of colorectal neoplasms in patients with non-alcoholic fatty liver disease: A systematic review and meta-analysis
Source: PLoS One. 2021 Jan 25;16(1):e0245921. doi: 10.1371/journal.pone.0245921 (PMC7833217; doi:10.1371/journal.pone.0245921)
Supplement: S1 Checklist — (PDF) [file pone.0245921.s001.pdf]

PRISMA 2009 Checklist

Section/topic	#	Checklist item	Reported on page #
**TITLE**			
Title	1	Site-specific risk of colorectal neoplasms in patients with non-alcoholic fatty liver disease: a systematic review and meta-analysis	1
**ABSTRACT**			
Structured summary	2	Background: Many studies have shown that NAFLD is indeed closely related to the occurrence of colon tumors.The aim of this study was to further establish an assessment for the risk associated with NAFLD and the site-specificity of colon tumors.<br>Methods: We searched the PubMed,Embase,Cochrane,and Scopus databases published from January 1,1981,to December 15,2019,assessing the risk of colorectal neoplasms in patients with NAFLD.The primary outcome measure was the incidence of site-specific risk of colorectal neoplasms in patients with NAFLD reported as ORs which pooled under a random-effects model and calculated via Mantel-Haenszel weighting.The study is registered with PROSPERO, number CRD42020162118.<br>Results: 11 articles（12,081 participants）were included in this meta-analysis.After heterogeneity removed,the overall risk-value pooled for right colon tumors(OR=1.60,95% CI 1.27-2.01,I2 =58%,P=0.02)was higher than the left(OR=1.39,95% CI 1.11-1.73,I2 =59%,P=0.02).However, this outcome was unclear when considering gender differences (Male&Right:OR=1.05; Male&Left:OR=1.26; Female&Right: OR=1.17; Female&Left:OR=1.17).The incidence of right colon tumors(Asian&Right:OR=1.56)was obviously higher in Asians with NAFLD than the left (Asian&Left:OR=1.23),while the risk relevance was similar and moderately associated with an increased risk of incident double-sided colorectal tumors in Europeans (European&Right:OR=1.47; European&Left:OR=1.41). The outcome of pathological morphology includes：the advanced adenoma OR=1.82;the tubular adenoma OR=1.24;the serrated adenoma OR=2.16.<br>Conclusions: NAFLD is associated with a high risk of colon tumors,especially in regard to tumors of the right colon, which are more prevalent in Asian populations.	2
**INTRODUCTION**			
Rationale	3	Hypertension, T2DM, hyperlipidemia and other metabolic syndromes, insulin resistance (IR), and obesity are important risk factors shared by the occurrence and progression of colon tumors and NAFLD. In 1998, the concept of a "hepato-intestinal axis" was proposed by Marshall, suggesting that the liver and intestinal tract maintain a similar anatomical structure and possess an acquired functional relationship to some extent. In patients with NAFLD, the increased levels of insulin-related factors, including insulin-like growth factor (IGF-1) and pro-inflammatory cytokines (TNF-α, IL-6, and IL-8), provide an ideal microenvironment for the growth of colonic mucosal tumors. Lower adipokines and higher leptin levels might also mediate NAFLD and carcinogenesis. Aadipokines regulate metabolism, inflammation, and fibrogenesis and can inhibit colon tumor growth via cyclic AMP-activated protein kinases, inducing caspase-dependent pathways, which are involved in endothelial cell apoptosis 7-11. Neoplasms (adenoma or cancer) at different sites of colon present significant heterogeneity at the clinical, histological, and molecular levels, and could be considered as distinct tumor entities. A large number of clinical observations have confirmed the relationship between NAFLD and colon neoplasms and its association with specific tumor locations.	4-6

Objectives	4	This current study aimed to establish an assessment of the risk associated with NAFLD and the site-specificity of colon tumors while also exploring the influence of gender,nationality, and polyp pathological morphology factors with the goal of promoting the early screening and prevention of colon tumors.	
**METHODS**			
Protocol and registration	5	The study is registered with PROSPERO, number CRD42020162118.	9
Eligibility criteria	6	Inclusion criteria were (a) published as an original article; (b) used a cohort or cross-sectional design; (c) reported the risk of colorectal neoplasms in patients with NAFLD, in terms of standardized risk as an odds ratio (OR) or relative risk (RR); (d) diagnosed by liver biopsy, imaging, or computed tomography (CT) from individuals in the absence of excessive alcohol consumption and other known causes (e.g., viral, drugs) of chronic liver diseases, and the diagnosis of colon tumor was based on either biopsy or colonoscopy techniques, reporting site-specific incidence of colonic tumors in detail (left and right colon or proximal colon and distal colon or sigmoid, transverse, descending, ascending colon, splenic flexure, rectum, and cecum).	6-7
Information sources	7	We searched the PubMed, Embase, Cochrane, and Scopus electronic databases for English language studies published from January 1, 1981, to December 15, 2019, assessing the risk of colorectal neoplasms in patients with non-alcoholic fatty liver disease	6
Search	8	("non-alcoholic fatty liver disease" or "fatty liver" or "non-alcoholic steatohepatitis" or "NAFLD") AND ("colorectal neoplasms" or "colorectal neoplasm" or "colorectal adenomas" or "colorectal adenoma" or "colorectal cancer" or "colorectal tumor" or "colorectal carcinoma" or "colonic neoplasms" or "colonic neoplasm" or "colonic adenomas" or "colonic adenoma" or "colonic cancer" or "colonic tumor" or "colonic carcinoma" or "colon cancer" or "adenomatous polyps").	6
Study selection	9	The literature search identified 2554 records, of which 2522 were excluded after duplicates were removed and an initial screening of titles and abstracts. A total of 32 full-text articles were assessed for eligibility, and all of the studies that did not mention specific sites of the colon tumors or report complete dates were excluded during this process. Finally, only 11 articles8,13,22-30 were included in the meta-analysis after further screening and discussion.	9
Data collection process	10	Two authors (Wang and Lin) independently extracted the following data from all of the eligible studies using a predefined data extraction form: study characteristics (authors, year, and study design), study setting (region), study population characteristics (sample size and other basic data), outcomes (percentage of NAFLD and no-NAFLD patients with left or right colonic tumors), and detailed adjusted factors for effect index.	7
Data items	11	The number of diarrhea in the experimental group and the control group and the sample size of each group.	7
Risk of bias in individual studies	12	The Ottawa scale (NOS)16was used as a quality evaluation tool, and selection, comparability, and outcome were gauged by assigning points based on the NOS values from 1 to 9. Studies with a rating of 6 or higher were considered of high quality17. Areas of discrepancies or uncertainty were resolved by consensus.	7
Summary measures	13	The primary outcome measure was the incidence of the site-specific risk of colorectal neoplasms in patients with NAFLD, reported as ORs and pooled under a random-effects model. The OR for each study was defined as the reported number of NAFLD patients diagnosed with left or right colorectal neoplasms compared with the number of no-NAFLD patients. The 95% confidence intervals were considered as the effect size for all of the eligible studies.	8

Synthesis of results	14	A random-effects analysis method was also used to combine studies into defined subgroups based on gender, pathologic tumor morphology, study design type, or nationality, thus assigning a relative weight to each study within the subgroup that summed to 100%. The relative weight of each study was calculated via Mantel-Haenszel weighting.We assessed heterogeneity across the studies using the I^2 statistic and Cochran's Q statistic. I^2<50% and P>0.10 indicated a substantial heterogeneity. The heterogeneity of the studies was minimized by adjusting the form of the effect model (e.g., replace the fixed effects model with a random-effects model) or the selection of the effect size (e.g., replace OR with RR) if there was obvious heterogeneity. Sensitivity analysis of each individual study in the pooled analysis results and subgroup influence analysis was performed, if necessary. The possibility of small-study effects (publication bias) was assessed across the studies using a funnel plot as well as Egger's and Begg's regression asymmetry tests.	8

Section/topic	#	Checklist item	Reported on page #
Risk of bias across studies	15	The Ottawa scale (NOS)16was used as a quality evaluation tool, and selection, comparability, and outcome were gauged by assigning points based on the NOS values from 1 to 9. Studies with a rating of 6 or higher were considered of high quality17. Areas of discrepancies or uncertainty were resolved by consensus.	7
Additional analyses	16	The analyzed data indicated that the significant heterogeneity between NAFLD and risk of incident colorectal tumors was consistent for both left and right colonic tumors.So sensitive analysis has to be done.Subgroups of study type, sex, and pathologic type were preprogrammed	8
**RESULTS**			
Study selection	17	The literature search identified 2554 records, of which 2522 were excluded after duplicates were removed and an initial screening of titles and abstracts. A total of 32 full-text articles were assessed for eligibility, and all of the studies that did not mention specific sites of the colon tumors or report complete dates were excluded during this process. Finally, only 11 articles8,13,22-30 were included in the meta-analysis after further screening and discussion.	9
Study characteristics	18	A total of 4 of the 11 studies reported the risk correlation relationship in regard to the gender specificity of patients with NAFLD suffering from left- or right-half colon tumors, with each gender involving three studies. From the pathological tumor morphology, four papers presented advanced tumors, two papers included tubular adenoma, and three papers included serrated adenoma. In terms of the study design type, there were four cross-sectional studies and five studies classified as longitudinal studies. The geographical distribution of these studies was relatively balanced, with five of them carried out in Asia and four studies carried out in Europe. Each of these classifications was included in the subgroup analyses.	14
Risk of bias within studies	19	The median Newcastle-Ottawa rating for the six studies included was 6.5, of which only one study received a quality rating below the defined high-quality research standard of 6 stars.	9
Results of individual studies	20	The overall risk-value pooled for right colon tumors(OR=1.60,95% CI 1.27-2.01,I2 =58%,P=0.02)was higher than the left(OR=1.39,95% CI 1.11-1.73,I2 =59%,P=0.02).However, this outcome was unclear when considering gender differences (Male&Right:OR=1.05; Male&Left:OR=1.26; Female&Right: OR=1.17; Female&Left:OR=1.17).The incidence of right colon tumors(Asian&Right:OR=1.56)was obviously higher in Asians with NAFLD than the left (Asian&Left:OR=1.23),while the risk relevance was similar and moderately associated with an increased risk of incident double-sided colorectal tumors in Europeans (European&Right:OR=1.47; European&Left:OR=1.41). The	2

		outcome of pathological morphology includes：the advanced adenoma OR=1.82;the tubular adenoma OR=1.24;the serrated adenoma OR=2.16.	
Synthesis of results	21	11 articles（12,081 participants）were included in this meta-analysis.After heterogeneity removed,the overall risk-value pooled for right colon tumors(OR=1.60,95% CI 1.27-2.01,I2 =58%,P=0.02)was higher than the left(OR=1.39,95% CI 1.11-1.73,I2 =59%,P=0.02).	14
Risk of bias across studies	22	The median Newcastle-Ottawa rating for the six studies included was 6.5, of which only one study received a quality rating below the defined high-quality research standard of 6 stars.	9
Additional analysis	23	The outcome was unclear when considering gender differences (Male&Right:OR=1.05; Male&Left:OR=1.26; Female&Right: OR=1.17; Female&Left:OR=1.17).The incidence of right colon tumors(Asian&Right:OR=1.56)was obviously higher in Asians with NAFLD than the left (Asian&Left:OR=1.23),while the risk relevance was similar and moderately associated with an increased risk of incident double-sided colorectal tumors in Europeans (European&Right:OR=1.47; European&Left:OR=1.41). The outcome of pathological morphology includes：the advanced adenoma OR=1.82;the tubular adenoma OR=1.24;the serrated adenoma OR=2.16..	15-18
**DISCUSSION**			
Summary of evidence	24	No formal guidelines or recommendations regarding routine cancer screening for colorectal neoplasms among patients with NAFLD have been established at this point, even though a substantial proportion of clinical observations and system analyses in this field have demonstrated their close correlation. The analyses in this current study have further clarified the risk of site-specific colorectal neoplasms in these patients, and the findings of this meta-analysis have provided further evidence to support the idea that the risk of colorectal neoplasms is increased among patients with NAFLD, especially tumors the stem from the right-side colon (OR=1.60). In view of the relatively few studies included in this analysis (n=11), it was hard to define the site-specific risk correlation between colorectal tumors and NAFLD. Instead, the specific pathological morphologies of the tumors were assessed, including advanced adenoma, tubular adenoma, and serrated adenoma. It is noteworthy that there were a significantly higher prevalence and incidence of advanced adenoma (OR=1.82) and serrated adenoma (OR=2.16) relative to the general population, which is consistent with the conclusion presented above. Tubular adenoma, more common in the left colon, presented at a lower detection rate in this analysis.	18
Limitations	25	Some limitations exist in this current study. First, due to the inherent limitations of retrospective cross-sectional studies (n=4), time-event studies are not available for further follow-up to obtain the true results regarding the possible future event incidence. Second, the distribution of the funnel plot and Begg’s test are symmetrical, and the vertical coordinate representing the sample size is mostly concentrated at the top, indicating that the sample size is considerable and there is almost no publication bias. However, the heterogeneity of the results is still large, which might result in a result bias caused by the small number of studies or some other unknown issues. Third, the specific mechanism of the risk of colonic neoplasia caused by NAFLD is not yet known; specifically, the biological mechanism by which a lateral effect influences the risk of site-specific colonic neoplasms still needs to be defined. Lastly, no significant gender distribution specificity was found in the current studies; however, considering the economic cost to patients, the acceptance and safety of universal screening via colonoscopy among patients with NAFLD will be necessary to clearly define the age and gender targets for the initial screening.	22
Conclusions	26	This systematic review and meta-analysis suggest that NAFLD is associated with a high risk of colon tumors, especially tumors of the right colon, which are more prevalent in Asian populations. These results recommend early colonoscopy screening for patients with NAFLD to reduce the prevalence and incidence of colon tumors. It is also	23

		suggested that coordinated treatment of cardiovascular, endocrine, and liver diseases should be implemented in a timely manner in patients with NAFLD. Moreover, the development of a healthy diet, exercise program, and other healthy habits is warranted.	
**FUNDING**			
Funding	27	This work was supported by the National Science Foundation of China [No. 81774284]; State Administration of Traditional Chinese Medicine of the People's Republic of China [No.2019XZZX-ZL006]; Chengdu University of Traditional Chinese Medicine [No.19LW10]	23

From: Moher D, Liberati A, Tetzlaff J, Altman DG, The PRISMA Group (2009). Preferred Reporting Items for Systematic Reviews and Meta-Analyses: The PRISMA Statement. PLoS Med 6(7): e1000097. doi:10.1371/journal.pmed1000097

For more information, visit: **www.prisma-statement.org**.
