# Supplementary material for: Site-specific risk of colorectal neoplasms in patients with non-alcoholic fatty liver disease: A systematic review and meta-analysis
Source: PLoS One. 2021 Jan 25;16(1):e0245921. doi: 10.1371/journal.pone.0245921 (PMC7833217; doi:10.1371/journal.pone.0245921)
Supplement: Attachment — Submitted filename: Response to Reviewers.doc [file pone.0245921.s002.doc]

Dear Editors:

On behalf of my co-authors, we thank you very much for giving us an opportunity to revise our manuscript, we appreciate editor and reviewers very much for their positive and constructive

comments and suggestions on our manuscript entitled “ Site-specific risk of colorectal neoplasms in patients with non-alcoholic fatty liver disease: a systematic review and meta-analysis” . (I D: PONE-D-20-25326 ).

We have studied reviewer ’ s comments carefully and have made revision which marked in yellow highlight in the paper. We have tried our best to revise our manuscript according to the comments. Attached please find the revised version, which we would like to submit for your kind consideration. The following is our modification plan：

1、The English name of [author affiliation](../../../../C:/Users/Administrator/AppData/Local/youdao/dict/Application/8.9.3.0/resultui/html/index.html#/javascript:;) has been redefined in recent months, and updated here.
2、Line 13: We defined OS the first time it is used.
3、Line 40: We respelled the word "adipokines".
4、Line 22-23: By referring to the comments of the editor, we introduced the concept of “Metabolic (dysfunction) associated fatty liver disease” into the background of NAFLD in the introduction.
5、Line 298-301: After discussion, we also agree that the term "for the first time" is not appropriate, so we rephrase it to more accurately reflect the assessment undertaken.
6、Line 324-326: dear editors, Maybe you think the [content](../../../../C:/Users/Administrator/AppData/Local/youdao/dict/Application/8.9.3.0/resultui/html/index.html#/javascript:;) under the tittle “Acknowledgements“ should be credited in the “conflicts” title, but in Line 318-319 we have made “Conflict of interest”, so we decided to delete the part of “Acknowledgements”.

We would like to express our great appreciation to you and reviewers for comments on our paper.

Looking forward to hearing from you.

Thank you and best regards.

Yours sincerely,

Qiao-ling Wang

Corresponding author:

Name: Qiao-ling Wang

E-mail:qiaoling86@126.com
